# Age-specific incidence of allergic and non-allergic asthma

**DOI:** 10.1186/s12890-019-1040-2

**Published:** 2020-01-10

**Authors:** Johanna Pakkasela, Pinja Ilmarinen, Jasmin Honkamäki, Leena E. Tuomisto, Heidi Andersén, Päivi Piirilä, Hanna Hisinger-Mölkänen, Anssi Sovijärvi, Helena Backman, Bo Lundbäck, Eva Rönmark, Hannu Kankaanranta, Lauri Lehtimäki

**Affiliations:** 1 Department of Respiratory Medicine, Tampere University Hospital, FIN-33521 Tampere, Finland; 2 Faculty of Medicine and Health Technology, Tampere University, Tampere, Finland; 3 Department of Respiratory Medicine, Seinäjoki Central Hospital, Seinäjoki, Finland; 4 Department of Respiratory Medicine, Vaasa Central Hospital, Vaasa, Finland; 5 Unit of Clinical Physiology, Department of Clinical Physiology and Nuclear Medicine, HUS Medical Imaging Center, Helsinki University Central Hospital and University of Helsinki, Helsinki, Finland; 6 Heart and Lung Center, Helsinki University Central Hospital, Helsinki, Finland; 7 Department of Public Health and Clinical Medicine, Umeå University, Umeå, Sweden; 8 Department of Internal Medicine, Krefting Research Center, Sahlgrenska Academy, University of Gothenburg, Gothenburg, Sweden; 9 Allergy Centre, Tampere University Hospital, Tampere, Finland

**Keywords:** Asthma, Allergic, Non-allergic, Adult, Child, Adolescent, Incidence, Prevalence, Onset, Age-specific

## Abstract

**Background:**

Onset of allergic asthma has a strong association with childhood but only a few studies have analyzed incidence of asthma from childhood to late adulthood in relation to allergy. The purpose of the study was to assess age-specific incidence of allergic and non-allergic asthma.

**Methods:**

Questionnaires were sent to 8000 randomly selected recipients aged 20–69 years in Finland in 2016. The response rate was 52.3% (*n* = 4173). The questionnaire included questions on e.g. atopic status, asthma and age at asthma diagnosis. Asthma was classified allergic if also a physician-diagnosed allergic rhinitis was reported.

**Results:**

The prevalence of physician-diagnosed asthma and allergic rhinitis were 11.2 and 17.8%, respectively. Of the 445 responders with physician-diagnosed asthma, 52% were classified as allergic and 48% as non-allergic. Median ages at diagnosis of allergic and non-allergic asthma were 19 and 35 years, respectively. Among subjects with asthma diagnosis at ages 0–9, 10–19, 20–29, 30–39, 40–49, 50–59 and 60–69 years, 70, 62, 58, 53, 38, 19 and 33%, respectively, were allergic. For non-allergic asthma, the incidence rate was lowest in children and young adults (0.7/1000/year). It increased after middle age and was highest in older age groups (2.4/1000/year in 50–59 years old).

**Conclusions:**

The incidence of allergic asthma is highest in early childhood and steadily decreases with advancing age, while the incidence of non-allergic asthma is low until it peaks in late adulthood. After approximately 40 years of age, most of the new cases of asthma are non-allergic.

## Background

Rackemann was the first to introduce the concept of extrinsic/allergic and intrinsic/non-allergic asthma in 1947 and thus described the first phenotypes of asthma [1]. Over recent decades, cluster analyses have confirmed that asthma is more of a heterogeneous disorder rather than just a single disease. Several phenotypes have been introduced in addition to the ones established 70 years ago [2–6], but differentiating the phenotypes in clinical practice can be challenging. One of the answers appears to be the age of asthma onset and subsequently the division into childhood/early-onset and adult/late-onset asthma [6, 7].

Childhood asthma is commonly associated with allergy [8, 9]. Large cohorts have shown allergic sensitization as a risk factor for development [10, 11] and persistence of asthma in childhood [12]. On the other hand, there is a lack of comprehensive studies on the relevance of allergy to adult asthma. Although allergic sensitization has been reported as a risk factor for asthma in adults [13, 14] and adult-onset asthma [15], adult asthma is more often non-allergic than allergic [7, 16]. Also, the rates of allergic sensitization in adult-onset asthma are mostly below 50% [15–17]. According to a recent Finnish cluster analysis, allergic asthma diagnosed in adulthood was often associated with respiratory symptoms already during childhood [5]. However, contradicting results do also exist and a U.S.-based study reported only a slight difference in allergic sensitization in early- and late-onset asthma (72 and 63% in subjects with asthma onset before and after 40 years of age, respectively) [18].

It appears that there is a lack of knowledge on the allergic and non-allergic phenotypes of adult-onset asthma and the relation between allergy and asthma onset age. Our aim was to study the association between asthma onset age and allergy by assessing age at diagnosis and age-specific incidence of asthma in adult asthmatics with and without allergic rhinitis in a population-based postal questionnaire study conducted in Finland.

## Methods

### Study design and population

**Fig. 1:**
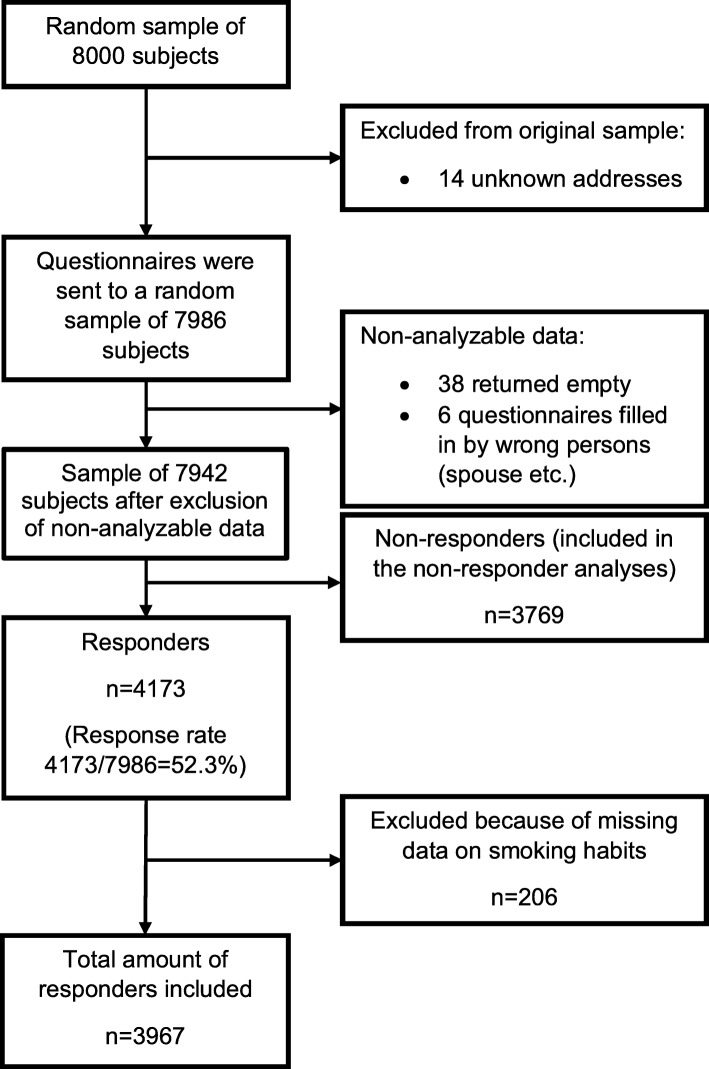
Study flow chart

The present study is part of the FinEsS (Finland-Estonia-Sweden) study, which is a postal questionnaire study on respiratory epidemiology conducted in collaboration in these three Northern European countries. Similar postal surveys were conducted in 1996, 2006 and 2016. The present study sample is part of the latest survey conducted in Finland in February 2016 and is formed from a random sample of 8000 subjects aged 20–69 years from the population in western Finland (Hospital Districts of Vaasa and Seinäjoki). The study sample was obtained from the Finnish Population Register and it was matched to the age and gender distribution of the population in the geographical area of our study. Finland is a bilingual country and the registered native language of a subject determined whether questionnaire in Finnish or Swedish language was used. The questionnaire was sent to a random sample of 7986 subjects after exclusion of subjects with unknown address. Two reminders were sent to those not responding. The sample size was 7942 subjects after further exclusion of subjects with non-analyzable data as shown in Fig. 1. In total, 4173 subjects responded yielding to a response rate of 52.3%. Of the responders, 206 were excluded because of missing data regarding smoking habits and thus, the actual sample size was 3967 responders included in the study. The study protocol was approved by the Ethical Committee of Helsinki University Hospital (approval number 200/13/03/00/15).

### Study area

The study area is mainly rural with two major towns (Seinäjoki and Vaasa, about 62,000 and 68,000 inhabitants, respectively). It has a subarctic climate and the yearly average temperature is 4 °C (from −7 °C in the winter to 17 °C in the summer) [19]. The most common allergic sensitizations in Finland are against dogs, cats and pollens whereas sensitization to house dust mites and molds is less common [20].

### Questionnaire and definitions

The FinEsS questionnaire was developed from the Obstructive Lung Disease in Northern Sweden (OLIN) questionnaire, which is modified from the Swedish translation of the British Medical Research Council (BMRC) questionnaire [21]. The questionnaire includes questions on respiratory diseases, symptoms, medication and comorbidities, risk factors and occupational factors considered relevant in respiratory epidemiology.

A physician-diagnosed asthma was defined by an answer “yes” to the question “Have you been diagnosed by a doctor as having asthma?”. Age at asthma diagnosis was determined by an answer to the question” What age were you when asthma was diagnosed?”. Allergic rhinitis was defined by an answer “yes” to either of the questions “Have you been diagnosed by a doctor as having allergic rhinitis caused by pollens (caused by e.g. birch, grass, mugwort)?” or “Have you been diagnosed by a doctor as having other allergic rhinitis (caused by e.g. cat or dog, but not pollen)?”. Allergic conjunctivitis was defined by an answer “yes” to the question “Have you been diagnosed by a doctor as having symptoms of allergy in your eyes?”. Age at diagnosis of allergy was not asked for. We used the presence and absence of allergic rhinitis as an indication of asthma being allergic or non-allergic, respectively. A sensitivity analysis was made by using the presence of either allergic rhinitis, allergic conjunctivitis or both as an indication of allergic asthma. Current smokers were considered those who reported smoking currently or during the 12 months preceding the survey. Ex-smokers reported previous smoking but had quit smoking at least 1 year prior to the survey. Never smokers did not report current or previous smoking.

### Statistical analysis

#### Reconstructing age-specific incidence of asthma from cross-sectional data

Incidence of asthma in different age groups was estimated based on cross-sectional data on current age of the responders and age at diagnosis of asthma [22, 23]. Longitudinal data were retrospectively reconstructed from the questionnaire data as if the 3967 subjects were a cohort of newborns recruited between 69 and 20 years ago. A “time-to-event” (age at diagnosis of asthma) was recorded for each individual, and the population at risk at each age was updated by subtracting both events (subjects reporting asthma diagnosed at younger age) and censorships (asthma-naïve responders younger than the age for which population at risk was calculated) from the total sample.

In brief, subjects were divided into 10-year age groups based on their current age, and annual incidence of asthma per 1000 person-years (new asthma diagnoses/1000/year) was calculated in each age group by dividing the number of incident asthmas in each group by age-group-specific population at risk, dividing the result by 10 and further multiplying with 1000. The 10-year age group -specific population at risk was a mean value of annually calculated respective 10-year risks. With respect to age 0, population at risk was all responders. For ages 1–20 years, subjects reporting asthma diagnosed at younger age than the age for which the population at risk was calculated were subtracted to form the 1-year population at risk. The youngest responders were 20 years of age at the time of the study. After age 20, the responders who did not report physician-diagnosed asthma (i.e. asthma-naïve responders) and who were younger than the age for which the population at risk was calculated, were also subtracted from all responders to calculate populations at risk for ages 21–69 years. Subjects reporting physician-diagnosed asthma but not the age at diagnosis were excluded from calculations.

#### Controlling for differences between older and younger age groups

When calculating incidence based on cross-sectional data, incidence rates at younger age represent means from several different age cohorts while incidence rates at older age represent those from older age cohorts only. Since different age cohorts may also have different overall incidence of atopy, relative proportion of allergic and non-allergic asthma may vary between different age cohorts and thus, might affect our estimates of early-onset and late-onset asthma. Therefore, we calculated separately in different age groups the proportion of allergic asthma among subjects with asthma diagnosed before the age of 40 years.

#### Statistical comparisons

Statistical analyses were performed using SPSS software version 23 (IBM Corporation, Armonk, NY) and 95% confidence intervals (CI) were calculated with EpiTools [24] by using the Wilson method. Mann-Whitney U test was used for continuous variables and Pearson chi-square test for categorical variables in comparisons between two groups. A *p*-value <0.05 was considered significant. Results are presented as percentages (95% CI) or medians (Interquartile range [IQR]).

## Results

### Characteristics of responders

Of the 7986 invited subjects, 4173 (52.3%) responded (Fig. 1). Basic characteristics of the responders are given in Table 1. Their median age was 53 years and a slight dominance of women was observed (52.2%). Among the 3967 responders included in the final analysis, 445 reported having a physician-diagnosed asthma yielding asthma prevalence of 11.2% (95% CI 10.3–12.2%). More in detail, 192 of 1898 men (10.1%; 95% CI 8.8–11.6%) and 253 of 2069 women (12.2%; 95% CI 10.9–13.7%) reported having asthma (*p* = 0.04) and the median age at asthma diagnosis was 21 (IQR 7–43) years in men and 29 (IQR 15–45) years in women (*p* = 0.03). Of the responders, 47.5% were either current or ex-smokers. Physician-diagnosed allergic rhinitis was reported by 7.2% of the subjects due to pollens only, by 3.1% due to other airborne allergens only and by 7.5% due to both of these, constituting 17.8% overall prevalence of allergic rhinitis (Table 1). Allergic conjunctivitis was reported by 11.7% of the subjects. The non-responder analysis of the study is published elsewhere [23].

**Table 1:** Characteristics of responders

Number of subjects	N	3967
Age^a^	Years	53 (38–63)
Female	N (%)	2069 (52.2)
BMI^b^	kg/m^2^	26.7 (4.9)
Smoking status	N (%)	
Current		798 (20.1)
Ex		1086 (27.4)
Never		2083 (52.5)
Physician-diagnosed asthma	N (%)	445 (11.2)
Allergic rhinitis	N (%)	706 (17.8)
No allergic rhinitis		3261 (82.2)
Allergic rhinitis due to pollen only		286 (7.2)
Allergic rhinitis due to non-pollen only		122 (3.1)
Allergic rhinitis due to pollen and non-pollen		298 (7.5)

^a^presented as median (IQR)

^b^based on responses of 3886 subjects and presented as mean (SD)

### Characteristics of responders with asthma according to presence of allergic rhinitis

Of the 445 responders with physician-diagnosed asthma, 230 (51.7%) had also allergic rhinitis (asthma considered as allergic) while 215 (48.3%) did not have allergic rhinitis (asthma considered as non-allergic) (Table 2). Non-allergic asthmatics were slightly older (58 vs 44 years, *p* < 0.001) and had a clearly older age at diagnosis of asthma (35 vs 19 years, *p* < 0.001) as compared to allergic asthmatics. In addition, subjects with non-allergic asthma had also slightly higher BMI (*p* = 0.046) and were more frequently ex-smokers compared to allergic asthmatics (*p* = 0.034) (Table 2).

**Table 2:** Characteristics of responders reporting physician-diagnosed asthma classified as allergic or non-allergic

		Responders with physician-diagnosed asthma and allergic rhinitis	Responders with physician-diagnosed asthma without allergic rhinitis	*p*-value
Total	N (%)	230 (51.7)	215 (48.3)	
Age^a^	Years	44 (32–59)	58 (39–66)	< 0.001
Female	N (%)	137 (59.6)	116 (54.0)	0.25
BMI^a^	kg/m^2^	25.7 (23.2–29.6)	27.4 (23.8–31.5)	0.046
Smoking status	N (%)			0.034
Current		42 (18.3)	46 (21.4)	
Ex		67 (29.1)	82 (38.1)	
Never		121 (52.6)	87 (40.5)	
Allergic rhinitis	N (%)			
Allergic rhinitis due to pollen only		64 (27.8)		
Allergic rhinitis due to non-pollen only		35 (15.2)		
Allergic rhinitis due to pollen and non-pollen		131 (57.0)		
Age at asthma diagnosis^a^	Years	19 (7–33)	35 (18–50)	< 0.001

^a^presented as median (IQR)

### Age-specific incidence of allergic and non-allergic asthma and their proportions

**Fig. 2:**
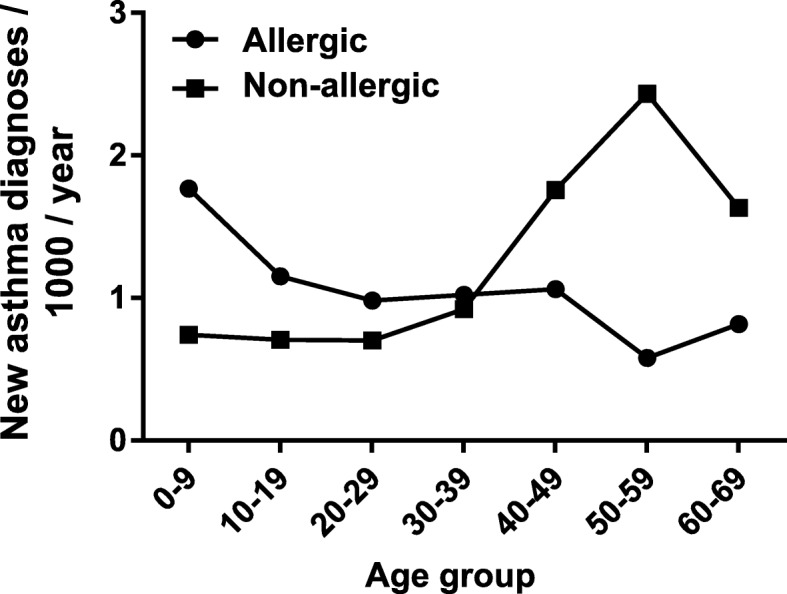
Incidence of new asthma diagnoses/1000 person-years divided into allergic (subjects with allergic rhinitis) and non-allergic (subjects without allergic rhinitis) cases in different age groups

Figure 2 shows incidence of allergic and non-allergic asthma in different age groups. A steady decline was observed in the incidence of allergic asthma with advancing age. The incidence of allergic asthma was highest in the youngest age group of 0–9 years (1.8/1000/year) and lowest in the age group of 50–59 years (0.6/1000/year). On the contrary, the incidence of non-allergic asthma was quite low and steady during childhood and early adulthood (about 0.7/1000/year) but it increased markedly after middle age and was highest (2.4/1000/year) in the age group of 50–59 year olds. Overall, the age-specific variation in incidence seemed to be higher for non-allergic than allergic asthma.

**Fig. 3:**
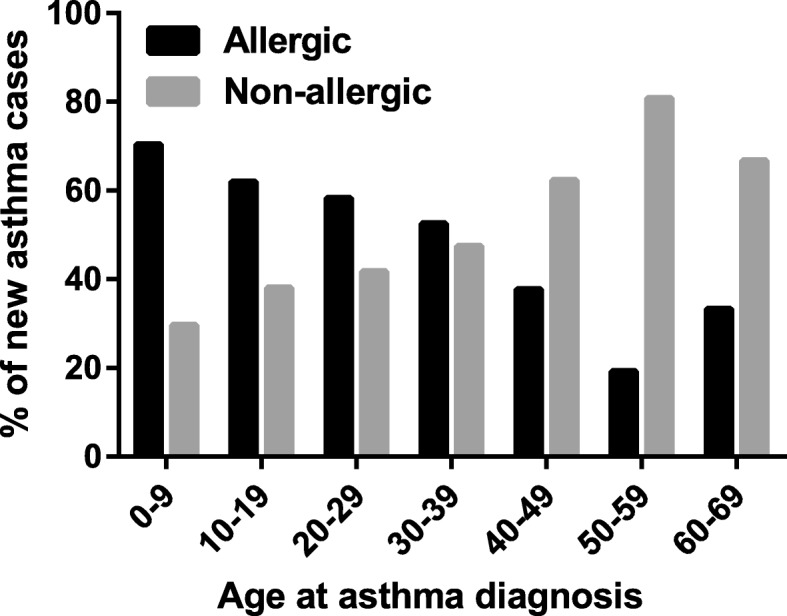
Relative proportions of allergic (subjects with allergic rhinitis) and non-allergic (subjects without allergic rhinitis) cases of new asthma diagnoses in different age groups

Figure 3 shows proportions of allergic and non-allergic asthma among new diagnoses of asthma in different age groups. In the age groups 0–9, 10–19, 20–29, 30–39, 40–49, 50–59 and 60–69 years, 70.4, 62.0, 58.3, 52.5, 37.7, 19.2 and 33.3% of the new asthma cases, respectively, were classified as allergic. More than 60% of the subjects with asthma diagnosed in childhood (< 18 years) reported having allergic rhinitis and thus were considered to have allergic asthma. Therefore, allergic asthma was the dominant phenotype as compared to non-allergic asthma until late twenties while non-allergic asthma became the dominant phenotype around the age of 40 and up to 80% of new cases of asthma were non-allergic in the older ages.

### Sensitivity analysis and controlling for possible cohort effect

To assess if the results depend on the definition of allergic asthma, a sensitivity analysis was performed and the results were quite similar when allergic conjunctivitis was included in the definition of allergy. Accordingly, among responders with asthma diagnosed at ages 0–9, 10–19, 20–29, 30–39, 40–49, 50–59 and 60–69 years, 72.4, 66.2, 63.3, 57.6, 47.8, 26.9 and 44.4% respectively, had either allergic rhinitis or allergic conjunctivitis or both (see Additional file 1: Figure S1). Incidence of non-allergic asthma was lowest in the younger ages (0.6/1000/year) and highest in the older age groups (2.2/1000/year in 50–59 years old) (see Additional file 2: Figure S2).

We found that in our sample prevalence of allergic rhinitis was lower in older age groups compared to younger age groups (prevalence of allergic rhinitis was 23.4, 27.2, 23.5, 15.6 and 10.5% in the age groups 20–29, 30–39, 40–49, 50–59 and 60–69 years, respectively, *p* = 0.036). To assess if the dominance of allergic cases among subjects diagnosed with asthma before the age of 40 years is dependent on the age cohort analyzed, we compared the proportions of allergic asthmatics among subjects diagnosed with asthma before the age of 40 years between three groups according to their current age: currently below 40 years of age, 40–60 years and over 60 years. Proportion of allergic asthma among asthma diagnosed before the age of 40 years was not statistically significantly different between the age groups (64% in subjects <40 years of age, 64% in subjects 40–60 years and 54% in subjects ≥60 years, *p* = 0.247).

## Discussion

We found that the incidence of allergic asthma was highest in childhood and gradually decreased in older age whereas the incidence of non-allergic asthma peaked in late adulthood. Most subjects with childhood-onset asthma were allergic while most subjects with asthma onset after 40 years of age were non-allergic.

Childhood-onset asthma is commonly associated with allergy [8, 9]. Our results supported this as close to 70% of responders with asthma diagnosed before 20 years of age had also allergic rhinitis in adulthood. It has been commonly recognized that asthma has its origin in childhood and the atopic early-onset asthma is the most important and widely recognized phenotype [25, 26]. However, a recent U.S.-based study showed adult-onset asthma as being the dominant phenotype among women in middle age [27]. In Finland during 2012–2013, 70% of new asthma diagnoses were made in adults indicating that adult-onset asthma is a clinically relevant phenotype [28]. Nevertheless, studies on adult-onset asthma are still scarce. In our study, the combined incidences of allergic and non-allergic asthma were highest after middle age and this was mainly driven by new cases of non-allergic asthma.

In the current study, the proportion of allergic asthma among new cases decreased steadily with advancing age at asthma diagnosis. There are some previous results that are in line with our finding, but in those studies age at asthma diagnosis was classified into two or three classes instead of looking at the prevalence of allergy on a wide range of asthma onset age classes [7, 29]. According to a European multi-center population-based study, atopy explained a minority (12–21%) of adult-onset asthma [17]. A Swedish and a Dutch study both reported prevalence rates of around 45% for atopy in adult-onset asthma [15, 16]. Warm et al. divided Swedish asthmatics into three age groups according to the age of asthma onset (≤ 6 years, 7–19 years and ≥ 20 years) and reported a decrease in the frequency of allergic sensitization in adulthood with increasing age of asthma onset (86, 56 and 26%, respectively) [29]. Our study results were similar as proportion of allergic rhinitis among new cases of asthma declined below 50% after the age of 35 years. To our knowledge this is the first study that shows the proportions of allergic and non-allergic asthma in consequent 10-year age groups at ages 0–69 years at asthma diagnosis.

Several studies using cluster analysis have also reported early-onset atopic asthma as a distinct phenotype [3, 6, 30]. The heterogeneity of phenotypes appears to increase with advancing age at asthma onset, resulting in the recognition of new phenotypes and risk factors especially in adult-onset asthma [5, 16, 31]. In our study, the responders with non-allergic asthma were more obese than allergic asthmatics. A phenotype of older, obese and less atopic women with frequent exacerbations and symptoms but at most only a moderate reduction in lung function has previously been reported [3, 5, 6]. Late-onset asthma is suggested to be more often non-allergic, severe and having a lower lung function than early-onset asthma [7, 32]. Accordingly, a cluster of severe late-onset less atopic asthma with eosinophilic inflammation and male-dominance has been identified [3]. A Finnish study with a follow-up of 12 years reported two adult-onset asthma phenotypes consisting predominantly of males [5]. The first cluster had non-atopic males with moderate smoking history who developed persistent airflow limitation at follow-up but with the lowest number of uncontrolled asthma. The other cluster contained older men with a heavy-smoking history, poor lung function and mostly uncontrolled asthma. Late-onset asthma phenotypes with milder clinical picture has also been reported such as a mild, untreated and less atopic adult-onset asthma [30] and a nonsmoking female-predominant cluster with good lung function and well-controlled/partially controlled asthma [5]. Generally thought, phenotypes that present more severe or symptomatic disease are especially identified in late-onset asthma [5, 6, 16, 33].

The reason for the decline in the incidence of allergic asthma with increasing age at asthma diagnosis may be related to at least two factors. Firstly, atopic allergy often begins in childhood and early adulthood while non-allergic asthma may be related to cumulative exposure to irritating factors such as occupational exposures and smoking and thereby becomes evident only later in life span after sufficient exposure times. Indeed, the higher proportion of ex-smokers among the non-allergic asthmatics in our study would support this hypothesis. Another reason may be the cohort effect that may affect incidence when estimated from cross-sectional data. Those subjects who have lived long enough to be able to have got late-onset asthma, were born earlier and represent different cohorts with lower overall prevalence of allergic sensitization than in younger generations. Moreover, according to population-based studies, the prevalence of allergic sensitization in general decreases with increasing age due to low incidence and higher remission [34]. Prevalence rates over 50% for allergic sensitization are reported among young adults compared to 26–39% in adults over 50 years of age [18, 20, 34]. A database search survey from the U.S. (2005–2006) showed a lower rate of allergic sensitization in asthmatics ≥55 years old when compared to 20–40 years old (65 and 75%, respectively) [18]. In the same survey, it was shown that if the analysis of asthma onset age and allergy is restricted to only subjects who were at least 55 years old, the difference in the frequencies of atopic sensitization between asthmatics with onset before or after 40 years of age was low (72% vs 63%, respectively), but the numbers of subjects were quite small (12 and 31, respectively) to make firm conclusions. A recent Swedish study reported a continued increase in the prevalence of allergic asthma in the last 20 years (from 5.0% in 1996 to 7.3% in 2016) [35], which may reflect the overall increase in the prevalence of atopic sensitization in new generations. Accordingly, in our sample the prevalence of allergic rhinitis was significantly lower in the oldest age cohort. However, in all age cohorts of our sample, asthma diagnosed before the age of 40 years was more often allergic than non-allergic, and the difference between age cohorts was not statistically significant in this regard. In the future, when the currently young generations with higher prevalence of atopy grow old, also late-onset asthma may have higher proportions of allergic cases.

In the present study, a responder was defined to have allergic asthma if he had both physician-diagnosed asthma and physician-diagnosed allergic rhinitis, but we did not have any objective tests on allergic sensitizations. According to a Swedish study, 83% of persons aged 21–40 years with an atopic tendency (production of IgE antibodies against any allergen) had allergic rhinitis, concluding that allergic rhinitis is a good marker for allergic sensitization and clinical allergy [29]. In another study, both allergic sensitization and allergic rhinitis were significantly associated with incidence of asthma in adulthood in bivariate analyses, but interestingly only allergic rhinitis was associated with asthma incidence in multivariate analysis [15]. This indicates that a clinical allergy is a better predictor of asthma than atopic sensitization, and allergic rhinoconjunctivitis doubles the risk for incident asthma among adults [36]. Furthermore, allergic conjunctivitis is often associated with allergic rhinitis and the term rhinoconjunctivitis is used [37]. This was also observed in the present study as the sensitivity analysis for allergic and non-allergic asthma in association with the age at asthma diagnosis did not noticeably change when allergic conjunctivitis was included into the definition of allergy (see Additional files 1 and 2). We did not take allergic skin diseases into account when defining allergy as the definition of allergic dermatitis/eczema is not as uniform as respiratory tract allergic diseases and poses a higher risk for misdiagnosis of allergy [37].

The present study was cross-sectional in nature and we did not specifically inquire whether the responder had allergic rhinitis or other allergy symptoms at the time of asthma diagnosis. For this reason, responders with asthma and allergic rhinitis diagnosed at some point of their life might not have had allergic rhinitis at the time of their asthma diagnosis. However, it has been reported that both allergic and non-allergic rhinitis usually precede asthma onset in children and adults [38, 39].

Study limitations also include that no objective measurements of lung function to confirm asthma diagnosis were obtained. In Finland, a person with persistent asthma is entitled to special reimbursements for expenses of asthma medication if he/she has an objectively confirmed variable expiratory airflow limitation or bronchial hyperresponsiveness as determined in international asthma diagnostic guidelines [40]. Therefore, most of the asthma diagnoses in Finland are based on lung function measurements and the reliability of the reported asthma diagnosis used in our study is probably high. There is a risk for recall bias as we requested a self-reported age at asthma diagnosis, which in Australia was estimated as most often inaccurate [41] while in Sweden it has been estimated as most often accurate [42].

## Conclusions

The incidence of allergic asthma is highest in early childhood and steadily decreases during adulthood, while the incidence of non-allergic asthma is low until it peaks in late adulthood. After 40 years of age most new cases of asthma are non-allergic. This study supports the concept that late-onset asthma is a separate entity and the mechanisms behind it differ from asthma beginning in childhood or in early adulthood.

## Supplementary information

**Additional file 1: Figure S1.** Relative proportions of allergic (subjects with either allergic rhinitis or allergic conjunctivitis or both) and non-allergic (subjects without allergic rhinitis or allergic conjunctivitis) cases of new asthma diagnoses in different age groups.

**Additional file 2: Figure S2.** Incidence of new asthma diagnoses/1000 person-years divided into allergic (subjects with either allergic rhinitis or allergic conjunctivitis or both) and non-allergic (subjects without allergic rhinitis or allergic conjunctivitis) cases in different age groups.

## Data Availability

The datasets used and/or analyzed during the current study are available from the corresponding author on reasonable request.

## Abbreviations

BMI: Body mass index
CI: Confidence interval
IQR: Interquartile range
SD: standard deviation
