# Supplementary figures and images for: Age-specific incidence of allergic and non-allergic asthma
Source: BMC Pulm Med. 2020 Jan 10;20:9. doi: 10.1186/s12890-019-1040-2 (PMC6954552; doi:10.1186/s12890-019-1040-2)

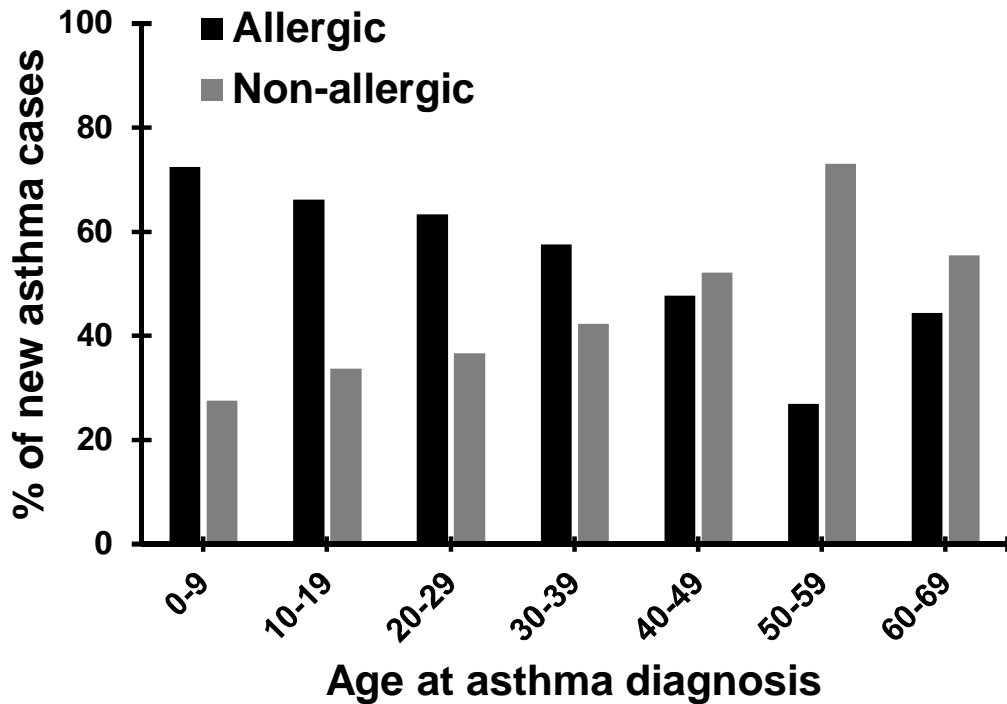

Supplementary Figure 1

Supplement: Supplementary file 1 — Additional file 1: Figure S1. Relative proportions of allergic (subjects with either allergic rhinitis or allergic conjunctivitis or both) and non-allergic (subjects without allergic rhinitis or allergic conjunctivitis) cases of new asthma diagnoses in different age groups. [file 12890_2019_1040_MOESM1_ESM.pdf]

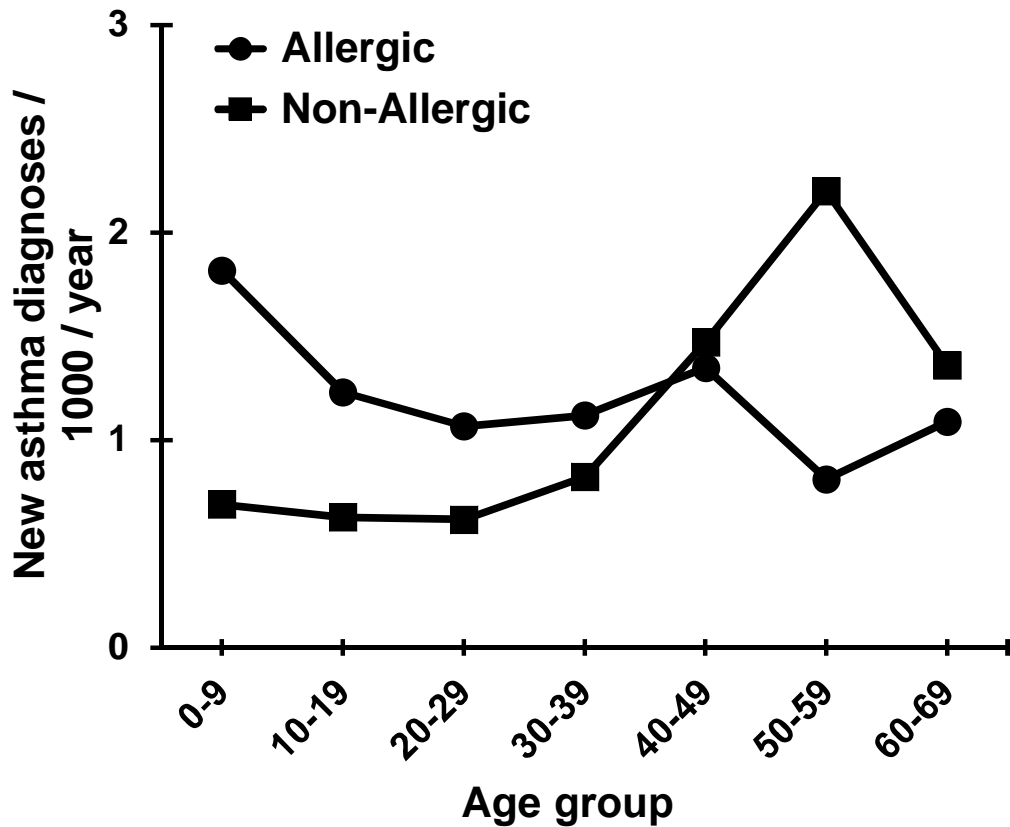

Supplementary Figure 2

Supplement: Supplementary file 2 — Additional file 2: Figure S2. Incidence of new asthma diagnoses/1000 person-years divided into allergic (subjects with either allergic rhinitis or allergic conjunctivitis or both) and non-allergic (subjects without allergic rhinitis or allergic conjunctivitis) cases in different age groups. [file 12890_2019_1040_MOESM2_ESM.pdf]
